# Innovation, evidence and uncertainty: rethinking the evaluation of neurovascular devices

**DOI:** 10.1093/esj/aakag092

**Published:** 2026-07-29

**Authors:** Christian A Taschner, Anne Christine Januel, Luca Valvassori, Johanna M Ospel, Jens Fiehler

**Affiliations:** Department of Neuroradiology, Medical Centre–University of Freiburg, Faculty of Medicine, University of Freiburg, Freiburg, Germany; Department of Neuroradiology, University Hospital Toulouse, Toulouse, France; Department of Neuroradiology, ASST Santi Paolo e Carlo, Milano, Italy; Department of Radiology and Clinical Neurosciences, University of Calgary, Calgary, Canada; Department of Diagnostic and Interventional Neuroradiology, University Medical Centre Hamburg-Eppendorf, Hamburg, Germany

**Keywords:** clinical evaluation, evidence standards, neurovascular devices, randomised controlled trials, real-world evidence

Volovici and colleagues raise a timely concern regarding the quality of evidence supporting intracranial aneurysm devices.^1^ The authors’ observation that many published studies suffer from methodological limitations, incomplete outcome reporting and insufficient long-term follow-up is well founded. Their call for greater transparency, independent outcome assessment, protocol registration and standardised outcome definitions deserves broad support.

However, the proposed solution—a general requirement for randomised controlled trials whenever alternative treatment options exist—reduces a complex methodological and regulatory problem to a single study design. Whether randomisation is appropriate depends not only on the presence of an alternative treatment, but also on device novelty, residual uncertainty, anticipated benefit, feasibility, disease prevalence, operator dependence and the regulatory objective.

Not all device innovations carry the same degree of uncertainty. First-in-class devices introducing new mechanisms of action differ fundamentally from incremental modifications of established technologies. Similarly, using an established technology in a new anatomical territory raises different evidence-related questions than testing a novel device. A universal requirement for randomised trials does not reflect these distinctions and risks ignoring them.

The methodological limitations identified by Volovici et al. do not imply that randomisation is the only solution. Methodological quality and randomisation are not synonymous. Prospective multicentre studies with consecutive enrolment, independent imaging adjudication, blinded clinical event committees, independent data monitoring, predefined statistical analysis plans, transparent reporting and robust post-market surveillance may provide highly informative evidence while remaining proportionate to residual uncertainty.

The characteristics of medical devices further limit the direct transfer of pharmaceutical evidence-generation paradigms. Unlike drugs, devices are frequently subject to iterative modifications, learning-curve effects, operator dependence and rapid technological evolution. Regulatory frameworks for devices therefore require a more nuanced approach than a rigid sequence of phase 2 and phase 3 randomised trials. Evidence generation should be proportionate rather than uniform. Applying a pharmaceutical-style development framework to medical devices overlooks these fundamental differences between drugs and devices.

Requiring sequential phase 2 and phase 3 randomised trials for a broad range of device modifications and indications may delay access to potentially beneficial technologies without necessarily improving evidence quality. In rapidly evolving fields such as neurointervention, prolonged evaluation pathways may simply not keep pace with technological innovation. A proportionate approach balances patient protection and timely access by matching evidentiary requirements to residual uncertainty, rather than applying uniform requirements across fundamentally different scenarios.

A further limitation concerns the process by which the proposed recommendations were developed. Despite potentially far-reaching implications for innovation, regulation, reimbursement and patient access, they are based on expert opinion rather than formal consensus methodology. No systematic evidence synthesis, Delphi process, stakeholder engagement procedure, predefined consensus thresholds or multidisciplinary consensus framework is reported. They should therefore be interpreted as expert opinion, not broadly endorsed methodological standards.

This distinction matters because alternative approaches have recently emerged that address a comparable problem through a more structured methodology. A recently published European multisociety consensus on clinical evidence standards for high-risk endovascular devices used in the treatment of ischemic stroke was informed by a scoping review and developed through a formal three-round Delphi process involving experienced neurointerventionalists and vascular neurologists from across Europe.^2^ Rather than prescribing a single study design, the resulting framework links evidentiary requirements to device novelty, residual uncertainty and regulatory objectives, while maintaining rigorous standards for study conduct, endpoint adjudication, data transparency and post-market evidence generation.

Importantly, this approach does not reject randomisation. Randomised trials remain essential when substantial uncertainty exists and clinically relevant differences between treatment strategies must be established. However, study design should be driven by the research question, residual uncertainty and regulatory objective. Randomisation should therefore be viewed as one methodological tool among several, rather than a universal prerequisite for device evaluation.

The challenge facing neurovascular device research is therefore not whether evidence should be rigorous, but how rigour can be achieved in a scientifically appropriate, clinically meaningful and realistic way. The future of device evaluation is unlikely to be served by a binary choice between randomised trials and observational studies. Instead, it will require a flexible methodological framework that matches study design to the evidence needed in each clinical and regulatory scenario.

In this respect, the debate initiated by Volovici and colleagues is valuable. Yet rather than asking whether all new devices should undergo randomised evaluation, the more relevant question may be: what level and type of evidence is most appropriate for a particular device, indication and degree of uncertainty? Only by addressing that question can we simultaneously protect patients, preserve scientific rigour and maintain feasible pathways for responsible innovation in neurovascular devices.
